# Highly Oriented SiC@SiO_2_ Ceramic Fiber Aerogels with Good Anisotropy of the Thermal Conductivity and High‐Temperature Resistance

**DOI:** 10.1002/advs.202416740

**Published:** 2025-03-06

**Authors:** Zheng Zhang, Cui Liu, Nian Li, Wei Guo, Ying Li, Pengzhan Yang, Shudong Zhang, Zhenyang Wang

**Affiliations:** ^1^ University of Science and Technology of China Hefei 230026 China; ^2^ Institute of Solid‐State Physics Hefei Institutes of Physical Science Chinese Academy of Sciences Hefei Anhui 230031 China; ^3^ The Key Laboratory of Photovoltaic and Energy Conservation Materials Hefei Institutes of Physical Science Chinese Academy of Sciences Hefei 230031 China

**Keywords:** anisotropy, high‐temperature insulation, SiC@SiO_2_ nanofiber aerogels, ultra‐low thermal conductivity

## Abstract

Here electrospinning and freeze‐drying techniques are combined to fabricate an anisotropic SiC@SiO_2_ ceramic fiber aerogels (A‐SiC@SiO_2_‐FAs). The anisotropic structure of the A‐SiC@SiO_2_‐FAs features aligned layers stacking layer‐by‐layer with the same direction and highly oriented 1D fibers inside each layer. The A‐SiC@SiO_2_‐FAs exhibit anisotropic thermal properties with an extremely low thermal conductivity of 0.018 W m^−1^ K^−1^ in the transverse direction (perpendicular to the SiC@SiO_2_ nanofibers) and ≈5 times higher thermal conductivity of 0.0914 W m^−1^ K^−1^ in the axial direction due to the highly oriented SiC@SiO_2_ nanofibers. The anisotropy factor of the A‐SiC@SiO_2_‐FAs is as high as 5.08, which exceeds most of the currently reported thermal insulation materials with anisotropic structural design, such as anisotropic wood aerogels, biaxially anisotropic PI/BC aerogels and anisotropic MXene foam, etc. The A‐SiC@SiO_2_‐FAs also have excellent thermal stability, maintaining structural integrity in oxidative environments at temperatures up to 1300^ °^C. Moreover, these structurally distinct A‐SiC@SiO_2_‐FAs result in superior elastic deformation with a radial recoverable strain exceeding 60% and an axial specific modulus of 5.72 kN m kg^−1^. These findings emphasize the potential of SiC nanofiber aerogels in extreme thermal environments and provide valuable insights for designing anisotropic insulation materials.

## Graphical abstract

The anisotropic‐structure SiC@SiO_2_ ceramic fiber aerogels feature aligned layers stacking layer‐by‐layer with the same direction and highly oriented 1D fibers inside each layer, resulting in an anisotropy factor as high as 5.08. Not only does it have 60% high recoverable deformation, but it also shows great promise for thermal protection in extreme environments (−196–1300^ °^C).

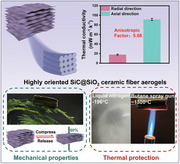

## 1 Introduction

Silicon carbide (SiC) aerogel has emerged as a promising material for thermal protection in key technological fields such as aerospace and military industries due to its low density, highly porous structure, thermal stability at high temperatures, and excellent chemical stability.^[^ ^1^, ^2^, ^3^, ^4^, ^5^, ^6^ ^]^Thereinto, SiC nanoparticle aerogels exhibit excellent thermal insulation properties due to their extremely high porosity and unique nano‐network structure,^[^ ^6^, ^7^, ^8^ ^]^ which effectively inhibit both gas‐phase and solid‐phase heat transfer. However, the high porosity and point‐to‐point rigid connections between 0D nanoparticles limit the deformability of these aerogels, resulting in low strength and high brittleness, which are prone to structural collapse and brittle fracture under complex mechanical loads.^[^ ^9^, ^10^, ^11^ ^]^ Recent research has demonstrated that the weak point‐to‐point connections between 0D nanoparticles can be effectively mitigated by introducing the continuous structure of 1D nanofibers, which helps overcome the brittleness of ceramic aerogels and enables elasticity and compressibility.^[^ ^12^, ^13^ ^]^ SiC nanowire‐reinforced ceramic aerogels, therefore, not only retain lightweight and high‐temperature resistance but also exhibit excellent recoverable deformation, significantly enhancing their mechanical properties.^[^ ^4^, ^11^, ^14^, ^15^, ^16^ ^]^ However, ceramic aerogels assembled from 1D fibers possess randomly distributed macropores (on the scale of tens of micrometers),^[^ ^13^, ^17^, ^18^, ^19^ ^]^ limiting their thermal insulation performance compared to nanoparticle aerogels. However, many conveniently conventional fabrication methods easily result in SiC nanowires/fibers forming randomly oriented, interconnected networks, leading to predominantly isotropic microstructures characterized by continuous solid conduction pathways.^[^ ^4^, ^17^, ^20^, ^21^, ^22^ ^]^ This structural arrangement may limit interfacial phonon scattering and attenuate the potential for effective thermal insulation.^[^ ^23^, ^24^, ^25^, ^26^ ^]^ Thus, the properties of aerogels are typically influenced by their intrinsic composition and internal structure, making rational composition control and structural design critical challenges for enhancing aerogel performance.

The layered structure and compositional design of materials often impart unique thermal properties. In nature, many biomaterials exhibit distinctive microstructures, such as natural wood. The “bottom‐up” growth of trees depends on vertically aligned woody channels responsible for the directional transport of water and nutrients.^[^ ^27^ ^]^ The hierarchical structure of nanocellulose and lignin endows nanowood with anisotropic mechanical and thermal insulation properties, where the thermal conductivity along the cellulose alignment is twice as high as in the perpendicular direction.^[^ ^26^ ^]^ Similarly, silkworm cocoons also demonstrate anisotropic properties, combining lightweight, toughness, and thermal insulation.^[^ ^28^, ^29^ ^]^ These properties are attributed to the naturally formed high‐strength, flexible silk and its laminated microstructure. The aligned silk layers provide the cocoon with a high Young's modulus and toughness, protecting the pupa from external impacts.^[^ ^29^ ^]^ Moreover, the anisotropic heat transfer within the laminated structure helps reduce heat flux perpendicular to the silk layers.^[^ ^28^ ^]^ This non‐uniform heat diffusion increases the heat flow in the direction of higher thermal conductivity while reducing it in the direction of lower thermal conductivity, enhancing the overall thermal insulation.^[^ ^4^, ^17^, ^26^, ^27^, ^30^, ^31^ ^]^ The greater the difference in thermal conductivity between directions, the more pronounced the insulation effect. As a result, anisotropic materials have significant potential in thermal management applications, particularly where directional heat transfer is needed. Examples include spacecraft insulation systems, heat dissipation in electronic devices, and directional conductive networks in battery electrodes, especially, where heat must be conducted along specific paths to minimize heat concentration elsewhere. Consequently, researchers have begun exploring the fabrication of anisotropic SiC nanofiber aerogels to enhance thermal insulation performance in extremely high temperatures. Su et al.^[^ ^17^ ^]^ developed anisotropic SiC nanowire aerogels by adjusting the suspension concentration and freeze‐drying conditions. The resulting aerogels exhibited a honeycomb‐like porous anisotropic structure with a framework of disordered, short SiC nanowires. Solid heat conduction parallel to the tubular pore direction exceeded gas‐phase conduction within the pores, resulting in an anisotropic thermal conductivity ≈2.5 times higher. In addition, due to crystallization‐induced chalking under large thermal gradients or high‐temperature exposure, the antioxidant resistance is insufficient thus exhibiting high‐temperature brittleness, and the crystallization inhibition and antioxidant resistance can be improved by adjusting the elemental composition and component structure of the ceramic materials.^[^ ^32^ ^]^ Studies have shown that the introduction of inorganic nanostructures such as silica, graphene, and nanodots can generate a large number of phonon barriers and significantly reduce the solid‐state thermal conductivity of the material.^[^ ^33^, ^34^, ^35^, ^36^ ^]^ For SiC nanofibers, an amorphous silica shell layer is introduced on their surface to form a stacking layer error resulting in forming a thermal conductivity barrier and slowing down the internal fiber oxidation rate to ensure superior thermal stability.^[^ ^17^, ^35^ ^]^ Therefore, the construction of anisotropic 1D nanofiber structures at the microscale can further reduce the thermal conductivity and achieve ultra‐high strength and thermal superinsulation properties.

Based on this concept, we first carefully picked SiC nanofibers with thermal stability at high temperatures and excellent chemical stability as aerogel units. Next, a thin amorphous SiO_2_ shell is grown on their surface, which acts as a thermal barrier, limiting phonon transport and further reducing the thermal conductivity of a single SiC nanofiber.^[^ ^17^ ^]^ Therefore, we employ electrospinning with a high‐speed orienter to produce highly oriented SiC nanofiber membranes, followed by heat treatment to introduce a thin amorphous SiO_2_ shell on their surface. Finally, a facile freeze‐drying technique to fabricate an anisotropic SiC@SiO_2_ ceramic fiber aerogels (A‐SiC@SiO_2_‐FAs), which featured aligned layers stacking layer‐by‐layer with the same direction and highly oriented 1D fibers inside each layer. The obtained aerogels achieved an ultra‐low thermal conductivity of ≈0.018 W m^−1^ K^−1^ in the direction perpendicular to the fibers and an anisotropy coefficient as high as 5.08. The A‐SiC@SiO_2_‐FAs also have excellent thermal stability, maintaining structural integrity in oxidative environments at temperatures up to 1300 °C. Moreover, the high degree of anisotropy allowed the aerogels to exhibit distinct mechanical properties in the axial and radial directions, with radial elastic deformation reaching up to 60%, and an axial specific modulus of up to 5.72 kN m kg^−1^. This study demonstrates that SiC@SiO_2_ aerogels prepared using this method are strong candidates for efficient thermal management applications and offer new insights for developing anisotropic thermal insulation materials.

## 2 Results and Discussion

### 2.1 Fabrication and Structure Characterization

**Scheme 1:**
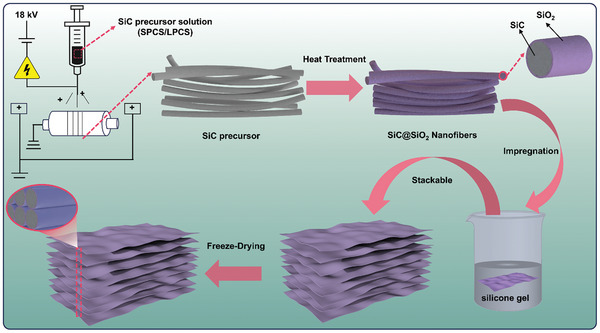
Preparation process of the A‐SiC@SiO2‐FAs.

This study presents a ceramic fiber‐based SiC@SiO_2_ aerogel with a highly anisotropic fiber arrangement and a layer‐by‐layer stacked structure, designed to achieve good anisotropy of the thermal conductivity, effectively high‐temperature thermal insulation, and robust mechanical properties. **Scheme** **1** illustrates an anisotropic SiC@SiO_2_ ceramic fiber aerogels (A‐SiC@SiO₂‐FAs) preparation process. First, the SiC fiber precursor was prepared by electrostatic spinning, then sintered with argon gas at 1450 °C to form SiC nanofiber film. Subsequently, an amorphous SiO_2_ shell layer is introduced on the surface of the SiC fibers by oxidation treatment. Finally, the SiC@SiO_2_ fiber film obtained was stacked layer by layer along the same direction and prepared into the desired aerogel by freeze‐drying.

To obtain the desired structure, we prepared SiC precursor fibers via electrostatic spinning of polycarbosilane solutions, using Liquid Polycarbomethylsilane (LPCS) and Solid Polycarbomethylsilane (SPCS) as reaction agents. In contrast to conventional electrostatic spinning devices, it should be pointed out that a high‐speed orienter (≈3000 rpm) was employed to assist nanofibers in forming parallel arrays on the receiver. The spun fibers experienced simultaneous effects from both the splitting electric field and the electrostatic force between like charges on the surfaces of the two grounded electrodes, causing the nanofibers to stretch across the gap perpendicularly to the edges of the electrodes. Accordingly, a SiC precursor fiber membrane exhibiting a highly oriented structure was successfully obtained (Figure S1a, Supporting Information), and the diameter of SiC precursor fiber was 500 nm (Figure S1b, Supporting Information).

**Figure 1:**
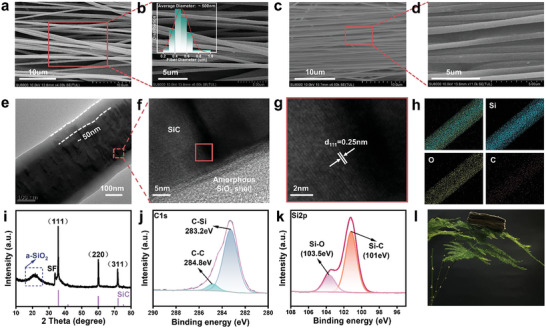
Characterization of the structure of the A‐SiC@SiO₂‐FAs. a, b) Highly oriented structure of SiC fiber membrane, and its local magnified SEM image. c, d) Highly oriented SiC@SiO_2_ fibrous membranes prepared by oxidation treatment and their localized enlarged SEM images. e) TEM images of single SiC@SiO_2_ fiber. f) Magnified high‐resolution TEM image of the labeled region in (e). g) High‐resolution TEM image of SiC crystalline phase. h) The corresponding EDS mapping of SiC@SiO_2_ fiber. i) XRD image of the A‐SiC@SiO_2_‐FAs. j, k) X‐ray photoelectron spectroscopy (XPS) images of a single SiC@SiO_2_ fiber membrane. l) Physical image of a piece of A‐SiC@SiO_2_‐FAs with a density of 27 mg cm^−3^ placed on a leaf of asparagus fern to demonstrate the lightweight nature of the aerogel.

In order to obtain the desired SiC fibers, the SiC precursor fiber membrane is pre‐oxidized at 80^ °^C to remove residual tetrahydrofuran (THF, boiling point 66^ °^C).^[^ ^37^ ^]^ The membrane is subsequently cured at 190^ °^C, which promotes surface oxidation and dehydrogenation reactions, resulting in the formation of Si─O and C═C groups (Figure S2, Supporting Information), enabling a continuous Si─C─Si cross‐linked network during high‐temperature heat treatment and preserving the fiber's structural morphology.^[^ ^38^, ^39^, ^40^ ^]^ Next, high‐temperature sintering was carried out in a tube furnace by passing argon gas to generate the crystalline phase SiC. Dehydrogenation and condensation reactions occur between groups in the PCS structure (the C─H and Si─CH_3_ bonds, Si─H and Si─CH_2_─Si groups) to release H_2_ and CH_4_, which form and increase the crosslinked Si─C─Si networks(Figure S3, Supporting Information).^[^ ^41^ ^]^ The fibers further pyrolyzed at 1000 and 1200 °C remain amorphous SiC fibers until the formation of a crystalline phase by pyrolysis at 1450 °C (Figure S4, Supporting Information). The scanning electron microscope (SEM) image in **Figure** **1**a shows the anisotropic arrangement of the SiC nanofiber membrane. As shown in Figure 1b, the SiC nanofibers with smooth surface and continuous fibers without breakage, and with a diameter of ≈500 nm (inset in Figure 1b), where desired oriented SiC fibers are successfully prepared.

Subsequently, a thin shell layer of amorphous SiO_2_ was introduced on the surface of the SiC fibers to form a thermal protection layer to enhance the thermal insulation properties of the aerogel. By controlling the temperature and holding time in the muffle furnace, the final oxidation conditions were determined to be 1000 °C for 30 min to prepare crystalline SiC‐ amorphous SiO_2_ core‐shell (SiC@SiO_2_) nanofibers structure (Figure S5, Supporting Information). From Figure 1c,d, it can be seen that the increase in the thickness of the amorphous SiO_2_ shell layer on the surface of the SiC fibers after the oxidation treatment is conducive to the close alignment of the fibers, which further enhances the anisotropic arrangement of the fibers and thus improves the structural stability of the fiber membrane. Moreover, due to the highly aligned fibrous structure, the SiC@SiO₂ fiber membrane exhibits an axial tensile strength ten times greater than its radial tensile strength, further demonstrating the successful fabrication of a highly aligned and continuous SiC@SiO₂ fiber membrane (Figure S6, Supporting Information). The successful construction of the amorphous SiO_2_ shell layer on the surface of the SiC fibers can be demonstrated by the transmission electron microscope (TEM) Figure 1e and the high‐resolution TEM image Figure 1f, and the thickness of the amorphous SiO_2_ shell layer is in the range of 50 nm (Figure 1e). It can be observed from Figure 1g that the lattice stripes are perpendicular to the fiber axis with a d spacing of 0.25 nm. The interfacial spacing corresponding to the 3C‐SiC (111) surface implies that the nanofibers grow along the [111] direction, and the energy dispersive spectroscopy (EDS) in Figure 1h also shows that fiber is mainly composed of the elements C, Si, and O. The relatively weak C mapping is attributed to the amorphous SiO₂ shell, which obscures the carbon signal from the internal SiC core.^[^ ^17^ ^]^ This is further confirmed by the X‐ray diffraction (XRD) pattern in Figure 1i. The main peaks correspond to the cubic 3C‐SiC structure, with peaks associated with the (111), (220), and (311) crystal planes at 2θ = 36°, 60°, and 72°, respectively, and the bulge in Figure 1i corresponds to the amorphous SiO_2_ peak (a‐SiO_2_). The small peak labeled “SF” before the strongest (111) peak is a small amount of stacking faults^[^ ^42^ ^]^ that accompanied the growth of SiC nanofibers and corresponds to the black shaded area in the selection of Figure 1f. The X‐ray photoelectron spectroscopy (XPS) indicates the successful preparation of SiC@SiO_2_ fibers (Figure 1j,k).

Finally, highly oriented SiC@SiO_2_ fiber membranes of specified sizes were fully impregnated in an aluminum borosilicate (AlBSi) binder and stacked layer by layer aligned in the same direction, rapidly cooled with liquid nitrogen, and then freeze‐dried to form the desired ultralight aerogel with a density of 27 mg cm^−3^. Figure 1l shows a physical picture of the prepared A‐SiC@SiO_2_‐FAs placed on a leaf of asparagus fern, demonstrating the lightweight nature of the aerogel.

### 2.2 Investigation of the Anisotropic Thermal Behavior of the A‐SiC@SiO_2_‐FAs

**Figure 2:**
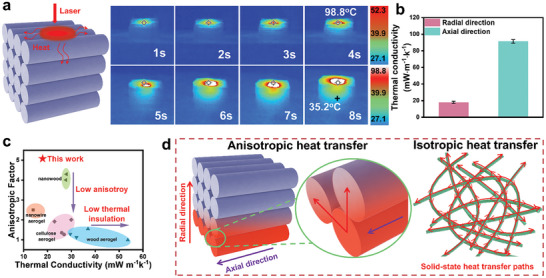
Efficient thermal insulating properties of the A‐SiC@SiO_2_‐FAs. a) Schematic diagram of the anisotropic heat transfer behavior of the A‐SiC@SiO_2_‐FAs explored by a laser light source and the infrared thermogram at the corresponding time. b) The A‐SiC@SiO_2_‐FAs Axial and radial thermal conductivity at room temperature. c) Plot of thermal conductivity vs anisotropy factor for our work and anisotropic aerogel‐like materials.^[^ ^17^, ^26^, ^27^, ^30^, ^31^, ^44^, ^45^, ^46^, ^47^, ^48^, ^49^ ^]^ d) Schematic of the mechanism of designing highly anisotropic structures to achieve efficient thermal insulation performance.

The heat transfer behavior of the A‐SiC@SiO_2_‐FAs was examined by observing heat flow along the axial and radial directions to test the anisotropic thermal behavior. As illustrated in **Figure** **2**a, applying a laser light source (radiation wavelength of 980 nm, power of 8 W, instantaneous temperature up to 300 °C) on top of the A‐SiC@SiO_2_‐FAs, the heat spot in the axial direction expanded rapidly and the heat increased at a rate of almost 25^ °^C s^−1^, reaching 98.8^ °^C after 4 s. In contrast, radial heat diffusion was slower, reaching only 35.2^ °^C in 8 s after twice the time. The anisotropy of the thermal conductivity is confirmed by the different thermal diffusion behaviors along the two main directions, which is similar to the heat transfer phenomena reported in the literature for thermally insulating materials with anisotropic structures.^[^ ^13^, ^26^, ^31^, ^43^ ^]^ And to further demonstrate the radial and axial thermal insulation effects, tests were conducted on a heating table with a fixed heat source at 200 °C. The prepared aerogel sample (20×20×20 mm) was placed on the heating stage, with temperature data collected from designated monitoring points. The results showed that the A‐SiC@SiO₂‐FAs placed along the radial direction after 20 min heating had better thermal insulation properties (Figure S7, Supporting Information). As shown in Figure 2b and Table S1 (Supporting Information), the room‐temperature thermal conductivity measured along the radial and axial directions is ≈18 and 91.4 mW m^−1^ K^−1^, respectively, with an anisotropy factor as high as 5.08, which is consistent with the observed anisotropic heat transfer behavior. Notably, this result significantly surpasses that of most currently reported thermal insulation materials with anisotropic structural designs (Figure 2c).^[^ ^17^, ^26^, ^27^, ^30^, ^31^, ^44^, ^45^, ^46^, ^47^, ^48^, ^49^ ^]^ Examples include biaxially anisotropic PI/BC aerogels (the anisotropy factor of 1.91),^[^ ^46^ ^]^ anisotropic CNTs@CoFe_2_O_4_/Polyimide aerogels (the anisotropy factor of 1.28),^[^ ^45^ ^]^ and anisotropic wood aerogels (the anisotropy factor of 4.29),^[^ ^27^ ^]^ which demonstrates the superiority of preparing anisotropic SiC@SiO_2_ ceramic fiber aerogels.

In particular, the anisotropically designed A‐SiC@SiO_2_‐FAs has a significantly lower thermal conductivity of 71.4 mW m^−1^ K^−1^ at 800 °C compared to the isotropic SiC@SiO_2_ nanofiber aerogel (Figure S8, Supporting Information). This is primarily due to the contributions of solid heat transfer and gas heat transfer in A‐SiC@SiO_2_‐FAs. As illustrated in Figure 2d, due to the disordered structure of the fibers constituting the isotropic aerogel units, the heat transfer paths are not oriented when heat is conducted along the fibers, thus exhibiting isotropic heat transfer behavior. The highly oriented structural design facilitates heat to be transmitted along the fibers and preferentially conduct in the axial direction and then diffuse in the radial direction, resulting in a hierarchical heat transfer. From a microscopic perspective, heat exhibits primary axial conduction and secondary radial diffusion along the fibers. Meanwhile, from a macroscopic perspective, the porous arch bridge structure between the layers of the fiber membrane mitigates solid heat transfer in the radial direction. Additionally, the construction of an amorphous SiO_2_ shell layer and SiC form a heterogeneous interface can effectively increase the interfacial thermal resistance and inhibit the heat transfer from fiber to fiber. This multifaceted synergistic effect leads to highly efficient thermal insulation properties in A‐SiC@SiO_2_‐FAs.

### 2.3 Fire‐Resistant and Chemically Stable Property

**Figure 3:**
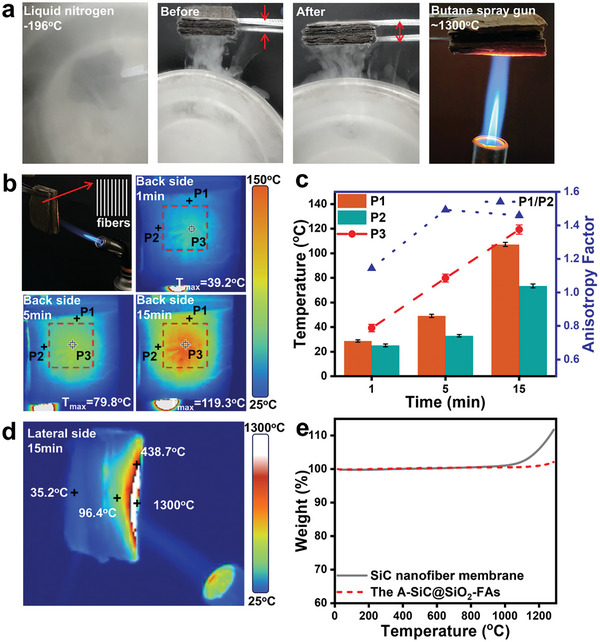
Thermal stability and low/high‐temperature resistance of the A‐SiC@SiO_2_‐FAs. a) Optical images of the A‐SiC@SiO_2_‐FAs under liquid nitrogen and butane gun treatment conditions. b) Optical and backside IR images of the A‐SiC@SiO_2_‐FAs exposed to a butane spray gun for 15 min. c) Temperature profiles and anisotropy factor (P1/P2) plots of observation points P1, P2 and P3 on the backside of the A‐SiC@SiO_2_‐FAs treated with butane torch for different time periods. d) Infrared thermography image of the lateral side of A‐SiC@SiO_2_‐FAs after exposure to a butane torch for 15 min. e) Thermogravimetric (TGA) curves of untreated SiC nanofiber membrane and the A‐SiC@SiO_2_‐FAs.

Besides the excellent anisotropic thermal insulation properties, we further investigated the practical performance and thermal shock resistance of the A‐SiC@SiO_2_‐FAs under low and high‐temperature extreme conditions. **Figure** **3**a and Movie S1 (Supporting Information) illustrate that the A‐SiC@SiO_2_‐FAs can return to its initial state under liquid nitrogen (−196 °C) by applying a certain pressure, in addition to the fact that no burning occurs when it is heated up in the flame of a butane blowtorch (≈1300 °C), which has been tested to prove that the A‐SiC@SiO_2_‐FAs can show sufficient structural stability. Furthermore, to investigate the thermal insulating behavior of the A‐SiC@SiO_2_‐FAs at sustained intense high temperatures, a 30×30×10 mm aerogel was taken and heated by a butane torch (Figure 3b). Real‐time observations were made using a thermal imager to measure the temperatures at the back and lateral sides of the aerogel. Figure 3b illustrates the optical image and fiber arrangement direction of the backside of A‐SiC@SiO_2_‐FAs continuously heated under a butane torch. After 15 min of heating, the maximum temperature at the backside (P3) reaches only 119.3 °C. It is also evident that the rate of heat diffusion at the center point is different along the axial (parallel to the fiber arrangement direction) and radial (perpendicular to the fiber arrangement direction). To quantify these observations, we measured the temperatures at points P1, P2, and P3 (center point) on the backside, as shown in Figure 3c, within 5 min, the heat diffusion rate from point P3 to point P1 was significantly greater than the rate from point P3 to point P2. The ratio of P1 to P2 is taken as the anisotropic diffusion factor, which further confirms that the A‐SiC@SiO_2_‐FAs also have an anisotropic heat‐transfer behavior at high temperatures. Moreover, after 15 min of continuous heating, the heat exposure on the lateral side of the aerogel was observed, as shown in Figure 3d. The maximum detectable temperature of the thermal imager is 550 °C; however, the lateral temperature near the backside of the aerogel measured only 35.2 °C. This result further emphasizes the slow diffusion of heat along the radial direction. Notably, no shrinkage or deformation of the aerogel was observed after 1 h of continuous heating with a butane flame (Movie S2, Supporting Information), highlighting the excellent refractory etch resistance of the aerogel.

We know that bare SiC nanowires and a thin SiO₂ layer (10–20 nm) encapsulating the surface are prone to further oxidation at high temperatures.^[^ ^17^ ^]^ However, our results showed that the amorphous SiO₂ shell layer provides good thermal protection for the SiC nanofibers(≈500 nm). In order to verify the thermal protective effect of the amorphous SiO_2_ shell layer, thermogravimetric analyses of the unoxidized SiC fiber membranes and the A‐SiC@SiO_2_‐FAs were performed. From the thermogravimetric curves in Figure 3e, it can be seen that the weight of the unoxidized SiC fiber membrane increased significantly after 1000 °C, indicating that the SiC fibers partially oxidized to form SiO_2_. While the weight of A‐SiC@SiO_2_‐FAs remained almost unchanged and showed excellent thermal stability. This is mainly because when the thickness of amorphous SiO₂ reaches a certain value (see Figure 1e, ≈50–60 nm), it can act as a protective layer, slowing down the oxidation rate of the SiC fibers inside, and thus improving the thermal resistance of the aerogel.^[^ ^20^ ^]^ Furthermore, Fourier transform infrared spectroscopy (FTIR) analysis of A‐SiC@SiO_2_‐FAs (Figure S9, Supporting Information) shows that the Si─C peak at 799 cm^−1^ is weakened while the Si‐O peaks at 440 and 1078 cm^−1^ are strengthened, which further supports the claim that the amorphous SiO_2_ layer can effectively inhibit the oxidation rate of the inner SiC fiber oxidation rate and has excellent thermal stability under high‐temperature thermal shock. The A‐SiC@SiO_2_‐FAs exhibit ultra‐low thermal conductivity and commendable thermal and chemical stability (1300 °C in an oxidizing environment and −196 °C at low temperatures), conferring significant advantages over conventional ceramic aerogels,^[^ ^2^, ^5^, ^49^, ^50^ ^]^ polymer‐derived aerogels,^[^ ^51^, ^52^, ^53^, ^54^ ^]^ commercial thermal insulation panels,^[^ ^55^ ^]^ and silicon‐carbide aerogels and their composites. This highlights the potential of the A‐SiC@SiO_2_‐FAs for applications at low/high‐temperature extremes.

### 2.4 Mechanical Properties of the A‐SiC@SiO2‐FAs

**Figure 4:**
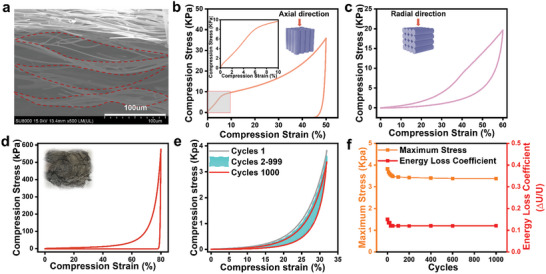
Compression cycle performance of A‐SiC@SiO_2_‐FAs. a) Microstructure of layered multi‐arch A‐SiC@SiO_2_‐FAs. b) Axial compressive stress‐strain curves of the A‐SiC@SiO_2_‐FAs and modeling diagrams. c) Radial compressive stress‐strain curves of the A‐SiC@SiO_2_‐FAs and modeling diagrams. d) Stress‐strain curves and physical diagrams of structural collapse of aerogels under maximum compressive stresses. e) Fatigue testing of aerogel along the radial direction for 1000 cycles with 30% compressive strain (ɛ). f) Energy loss coefficient and maximum stress vs number of cycles of compression.

Along with possessing excellent anisotropically thermal insulation properties and outstanding thermal stability at low/high temperatures. The A‐SiC@SiO_2_‐FAs obtained by stacking and freeze‐drying use AlBSi as a high‐temperature binder to form cross‐linked ceramic nanofiber networks. The cross‐linking activity arises from the silicate bonds (X─Si─O) formed between oxygen and nanofibers during calcination.^[^ ^56^ ^]^ During the freeze‐drying process,^[^ ^57^, ^58^ ^]^ ice crystals in the pores are replaced resulting in a porous structure. **Figure** **4**a displays the SEM micrographs of the aerogel, showcasing a hierarchically porous architecture characterized by interconnected layers and multi‐arched microstructures. As demonstrated in Figure S10a (Supporting Information), the amorphous SiO₂ phase promotes interfacial fusion between adjacent SiC nanofibers. Furthermore, the layer‐by‐layer stacking strategy using high‐temperature adhesive proves to be an effective approach for fabricating 3D layered fiber aerogels with structural stability.^[^ ^58^, ^59^, ^60^ ^]^ The 90° peel test confirmed a 10 kPa increase in the tensile strength of the A‐SiC@SiO_2_‐FAs interlayers fabricated by AlBSi bonding, which will contribute to the structural stability of the aerogel under complex stresses (Figure S10b, Supporting Information). Unlike the traditional method of breaking ceramic fiber membranes and re‐freeze‐drying them, the preparation of ceramic nanofiber aerogels with orientable structures is made simple, efficient, and controllable by stacking highly oriented fiber membranes prepared by electrostatic spinning layer‐by‐layer along the same direction and then freeze‐drying them. The prepared A‐SiC@SiO_2_‐FAs can be sheared into any desired shape, such as a rectangle, heart, circle, etc., in contrast to the brittle nature of traditional ceramic materials (Figure S11, Supporting Information).

Due to the anisotropy of the nanofiber membrane and the structure of the interlayer arch bridge, the A‐SiC@SiO_2_‐FAs exhibit strong anisotropic resistance to mechanical deformation, and through elastic deformation when subjected to external stress in the radial direction. The A‐SiC@SiO_2_‐FAs of 40×40×20 mm were selected for quasi‐static compression tests, and significant deformation behaviors were observed in both axial and radial directions. Figure 4b shows the stress–strain (σ–ε) curve under 50% axial strain. As with other ceramic fiber aerogels composed of randomly oriented flexible building blocks, three distinct phases are observed during loading: a linear elastic region for ε < 7%, a non‐linear elastic phase between 7% and 10% strain, and a densification phase beyond 10% strain. The compressive modulus (E) calculated from the linear elastic region is ≈154.39 kPa, resulting in a specific modulus (E/ρ) of ≈5.72 kN m kg^−1^. This is significantly higher than that of typical ceramic aerogels, such as hexagonal boron nitride aerogels (the specific modulus of ≈5),^[^ ^2^ ^]^ TiO_2_ nanofiber sponges (the specific modulus of ≈1.44),^[^ ^19^ ^]^ SiC nanowire aerogel (the specific modulus of ≈4.12),^[^ ^11^ ^]^ and SiO_2_ nanofiber aerogel (the specific modulus of ≈2.1).^[^ ^13^ ^]^ In contrast to the axial direction, the aerogel exhibits good recoverable deformation mainly in the radial direction, and it was tested that A‐SiC@SiO₂‐FAs could recover from 60% strain to the initial state after radial stress release (Figure S12, Supporting Information). The stress–strain (σ–ε) curve at a radial strain of 60% is shown in Figure 4c, where the nonlinear elastic behavior occurs around 35%. Which is inextricably linked to the multilayered and multi‐arch structure of the aerogels (Figure 4a). Figure S13 (Supporting Information) presents a schematic illustration of the compression process of the A‐SiC@SiO₂‐FAs. The diagram shows the arch bridge structure flattening under applied stress, with the fiber network returning to its original shape upon stress release. Specifically, the surface contact mode of the 2D nanofiber membranes used to construct the aerogel effectively alleviates stress concentration under applied stress.^[^ ^60^ ^]^ Furthermore, the multi‐arch bridge structure between fiber layers provides greater strain space when the aerogel is subjected to applied stress, thus improving the overall structural stability.^[^ ^61^ ^]^ However, when the strain reaches 80%, the collapse of the aerogel structure (Figure 4d) is mainly due to the inability of the arch‐bridge‐like structure in the aerogel to withstand the excessive stress (≈577 kPa), which leads to the fragmentation of the aerogel. (inset in Figure 4d). This demonstrates the anisotropic mechanical properties of the A‐SiC@SiO_2_‐FAs. Besides, cyclic compression tests were performed on the aerogel radial direction. Figure 4e presents the results of 1000 loading‐unloading fatigue cycles at 30% strain and a loading rate of 2 mm min^−1^. After 1000 cycles, the aerogels maintained their macroscopic shape with only slight permanent deformations of less than 5%. Figure 4f shows the energy loss coefficient as a function of maximum compressive stress over the number of cycles. After 1000 cycles, the energy loss coefficient stabilized at 0.11, demonstrating the excellent structural stability of the A‐SiC@SiO_2_‐FAs under cyclic fatigue testing.

## 3 Conclusions

In summary, we have successfully prepared highly anisotropic SiC@SiO_2_ nanofiber aerogels by electrostatic spinning technology and heat treatment process combined with freeze‐drying technology. The aerogel exhibits unique anisotropic properties, including an ultra‐low thermal conductivity of ≈0.018 W m^−1^ K^−1^ along the radial direction (perpendicular to the fiber membrane) at room temperature, with an anisotropy factor of 5.08, which is attributed to the highly anisotropically aligned nanofibers acting as an axial heat transfer channel, which enhances the axial heat transfer and thus reduces the radial heat transfer efficiency. Good structural and high‐temperature stability was demonstrated both at the very low temperature of liquid nitrogen and the intense high temperature of the butane torch. Second, the anisotropic self‐assembly strategy and laminated structure resulted in 60% elastic deformation in the radial direction, only 5% plastic deformation under 1000 cycles of compression, and good axial stiffness (5.72 kN m kg^−1^). This study demonstrates the great potential for performance enhancement of ceramic fiber aerogels through micro‐macrostructural modulation strategies, providing a reference for their application under extreme conditions and a new idea for the development of anisotropic materials.

## 4 Experimental Section

### Materials

Polycarbomethylsilane (PCS, Mw = 1000–2000), liquid polycarbomethylsilane (LPCS, Ceramic Conversion Rate:68%). The above chemical reagents were provided by Zhengzhou Alpha Chemical Co., Ltd., and aluminum chloride hexahydrate (AlCl_3_·6H_2_O, AR, 97%), boric acid (H_3_BO_3_, AR, ≥99.5%), polyvinylpyrrolidone (PVP, K90), tetrahydrofuran (THF, Anhydrous ≥99.9%) and anhydrous ethanol. The above chemical reagents were provided by Shanghai Aladdin Biochemical Technology Co., Ltd., and tetraethyl orthosilicate (TEOS, AR) was produced by Sinopharm Chemical Reagent Co., Ltd. None of the chemical reagents were further purified, and deionized water used in the experiments was prepared by the laboratory deionized water machine.

### Synthesis of Aluminum Borosilicate Binder (AlBSi)

The AlBSi binder was prepared by adding 1.95 g of AlCl_3_·6H_2_O, 0.1 g of H_3_BO_3,_ and 7.6 g of TEOS to 100 ml of deionized water and mixing for 15 min.

### Fabrication of the A‐SiC@SiO_2_‐FAs

To prepare SiC fibers, PCS and LPCS were dissolved in THF in a 3:2 mass ratio and stirred for 30 min at room temperature to form an 8 wt.% solution of SiC precursor, referred to as solution A. Similarly, PVP was dissolved in anhydrous ethanol at room temperature and stirred for 30 min to form a 12.5 wt.% solution of PVP, referred to as solution B. Then, the electrospinning precursor solution was prepared by mixing solution A and solution B in a volume ratio of 6:4 and stirring at room temperature for 8h. The resulting clear, transparent solution was transferred into a 10 ml plastic syringe containing a 25G spinning needle. Finally, the spinning was carried out using an electrospinning device, in which the room temperature was maintained at 25±2 °C, the relative humidity was 30±3%, the voltage was 18 kV the tip of the needle (High‐speed orientation receiver with a rotational speed of 2800r min^−1^, and 0.17 mm min^−1^ the constant feed rate) and the receiving distance was 15 cm. The collected fiber membranes were placed in an oven at 80 °C for 24 h to allow complete evaporation of the solvent. In order to obtain the desired structure during pyrolysis at high temperatures, the membranes were pre‐oxidized in an oven at 190 °C for 2 h to cure the fibers. Subsequently, the pre‐oxidized fibers were held at 1450 °C for 2 h at a heating rate of 2 °C min^−1^ under an argon atmosphere to obtain the desired highly oriented SiC fiber membrane. Finally, in order to introduce a thin amorphous SiO_2_ shell layer on the surface of the SiC fibers, the SiC fiber films were annealed in a muffle furnace at 1000 °C for 30 min to obtain SiC@SiO_2_ fiber films. Finally, highly oriented SiC@SiO_2_ fiber membranes of specified sizes were fully impregnated with aluminum borosilicate (AlBSi) binder and stacked layer by layer in the same direction, rapidly cooled with liquid nitrogen, and then freeze‐dried to form the desired aerogels.

### Characterizations

The microstructure of the A‐SiC@SiO_2_‐FAs was observed by scanning electron microscopy (SEM, SU8020, Hitachi, Japan) and transmission electron microscopy (JEM‐2010). Individual A‐SiC@SiO_2_‐FAs heat‐treated at different temperatures were characterized by XRD (XRD, X'Pert, PANalytical, Netherlands). The data of X‐ray photoelectron spectroscopy (XPS) were collected by a Thermo Scientific ESCALAB 250 high‐performance electron spectrometer. Fourier transform Infrared spectra (FT–IR) were conducted with Nicolet iS5 FTIR spectrometer. The thermogravimetric analyzer (TGA) was carried out on a simultaneous thermal analyzer (TGA, SDT650, TA Discovery, USA) and the samples (2–4 mg) were heated from room temperature to 1300 °C at a heating rate of 25 °C min^−1^ under an air atmosphere. The thermal conductivity of the A‐SiC@SiO_2_‐FAs was measured using a Laser flash apparatus (LFA 457, NETZSCH, Germany), and an infrared thermal camera (Fluke TIs75, USA) was used to document the thermal management properties of the aerogel material. Characterization of the aerogel (40 mm×40 mm×20 mm) an electronic universal testing machine (AGS‐X 10N‐10KN, Japan) was used for compression testing of layer‐stacked A‐SiC@SiO_2_‐FAs.

## Conflict of Interest

The authors declare no conflict of interest.

## Supporting information



Supporting Information

Supplemental Movie 1

Supplemental Movie 2

## Data Availability

Research data are not shared.
