# Supplementary material for: Highly Oriented SiC@SiO2 Ceramic Fiber Aerogels with Good Anisotropy of the Thermal Conductivity and High‐Temperature Resistance
Source: Adv Sci (Weinh). 2025 Mar 6;12(17):2416740. doi: 10.1002/advs.202416740 (PMC12061319; doi:10.1002/advs.202416740)
Supplement: Supplementary file 1 — Supporting Information [file ADVS-12-2416740-s003.docx]

**Supporting Information**

**Highly oriented SiC@SiO_2_ ceramic fiber aerogels with good anisotropy of the thermal conductivity and high temperature resistance**

*Zheng Zhang^1, 2^, Cui Liu^2, 3^, Nian Li^2, 3^, Wei Guo^2, 3*^, Ying Li^1,2^, Pengzhan Yang^1,2^, Shudong Zhang^2, 3*^, Zhenyang Wang^2, 3*^*

^1^ University of Science and Technology of China, Hefei 230026, China

^2^ Institute of Solid-State Physics, Hefei Institutes of Physical Science, Chinese Academy of Sciences, Hefei, Anhui, 230031, China

^3^ The Key Laboratory of Photovoltaic and Energy Conservation Materials, Hefei Institutes of Physical Science, Chinese Academy of Sciences, Hefei, 230031, China

*Corresponding author: Zhenyang Wang [(zywang@iim.ac.cn),](mailto:(zywang@iim.ac.cn),) Shudong Zhang (sdzhang@iim.ac.cn), Wei Guo (weiguo@issp.ac.cn)


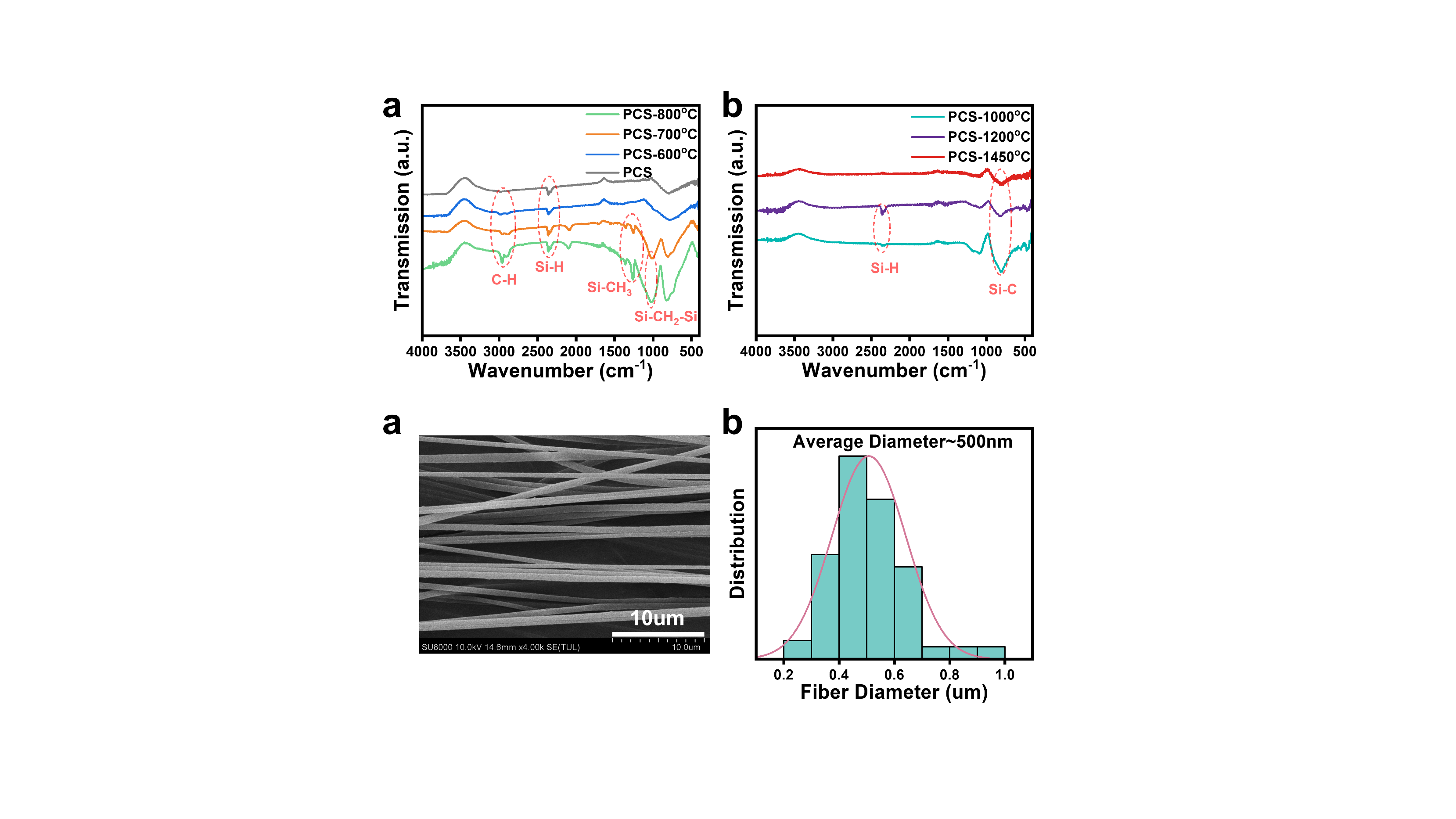


**Figure S1.** a) Highly oriented structure of SiC precursor fiber membrane, b) Average diameter of SiC precursor fiber.

**Figure S2.** Comparative infrared spectra of SiC precursor fibers, pre-oxidized fibers at 80 ^o^C and cured fibers at 190 ^o^C.


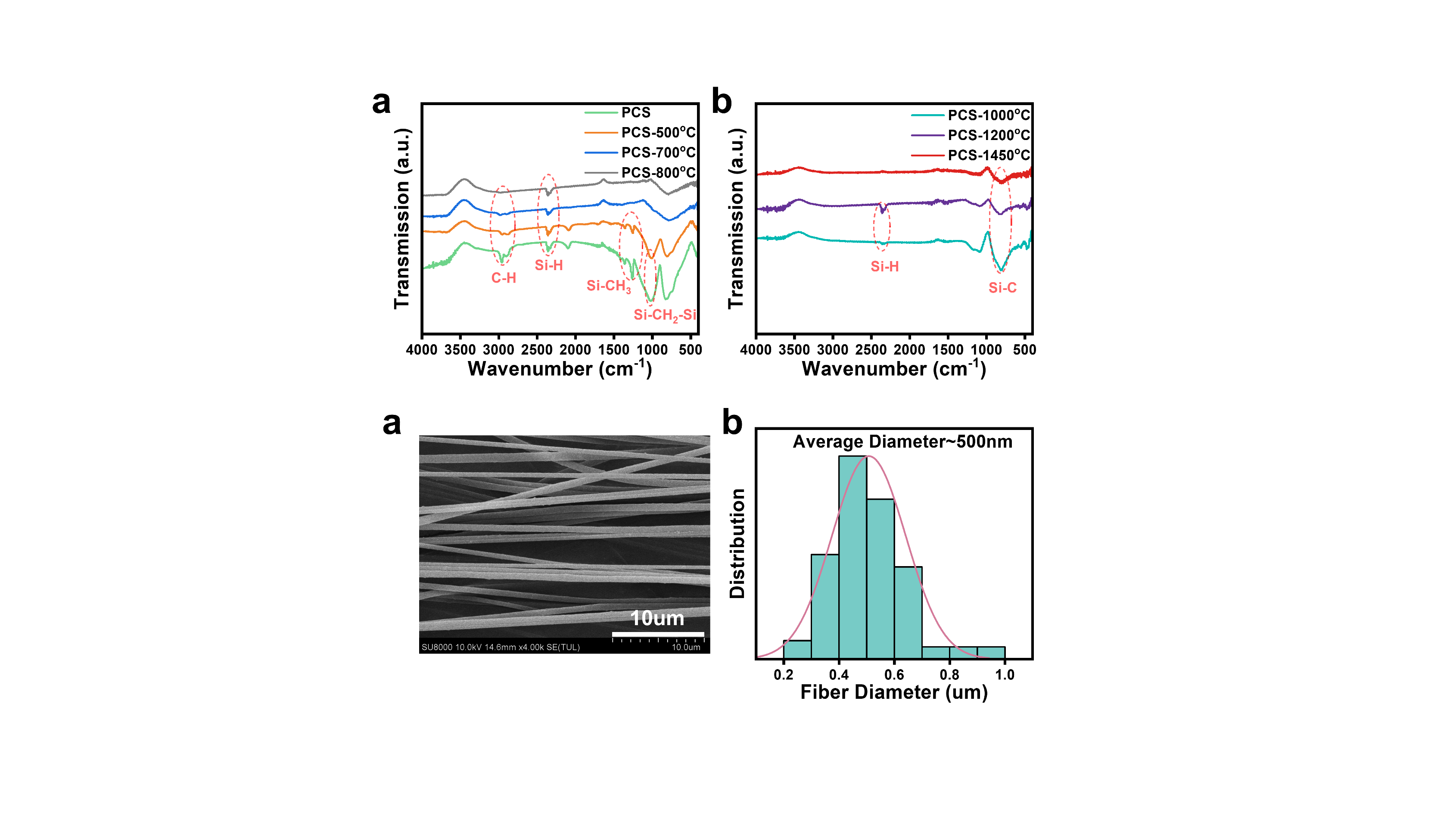


**Figure S3.** Infrared spectra of the decomposition of the SiC reaction agent polycarbosilane (PCS) at different temperatures.

**Figure S4.** SiC precursor fiber membrane sintered at different temperatures to achieve amorphous to crystalline phase transition.


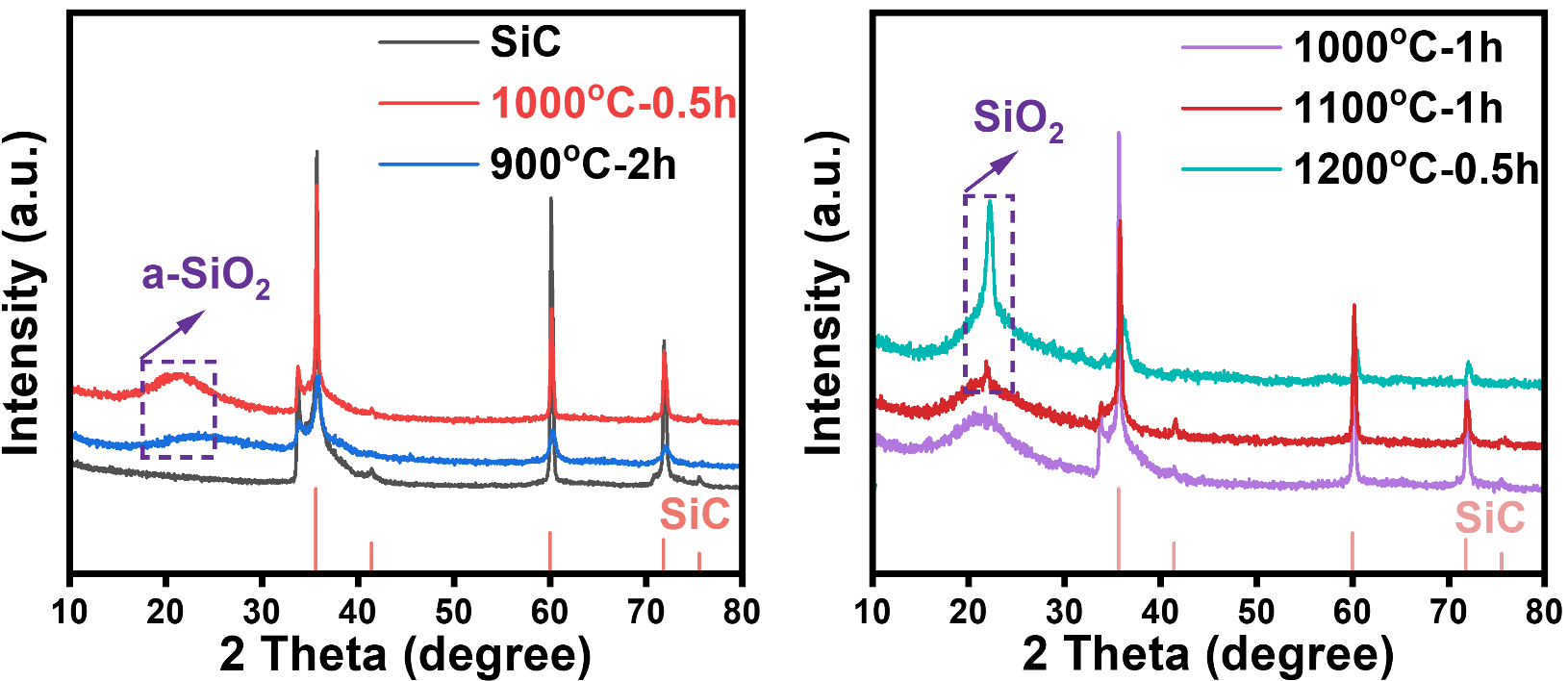


**Figure S5.** Control of the generation of amorphous SiO_2_ (a-SiO_2_) shell layer on the surface of SiC fibers, XRD pattern from a-SiO_2_ to crystalline SiO_2_ at different temperatures and times.


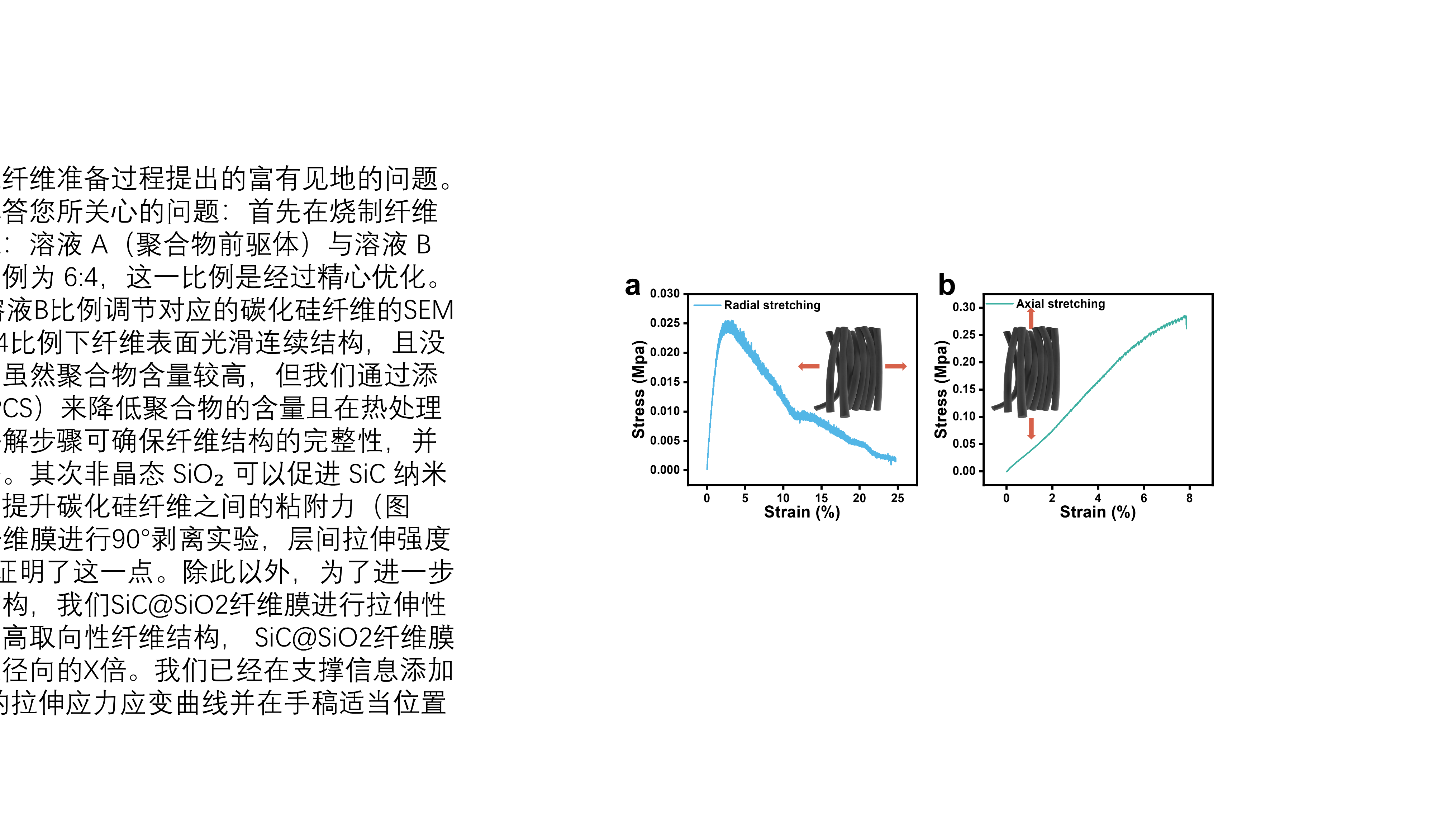


**Figure S6.** (a) The single SiC@SiO₂ nanofiber membrane radial tensile stress-strain curves. (b) The single SiC@SiO₂ nanofiber membrane axial tensile stress-strain curves.


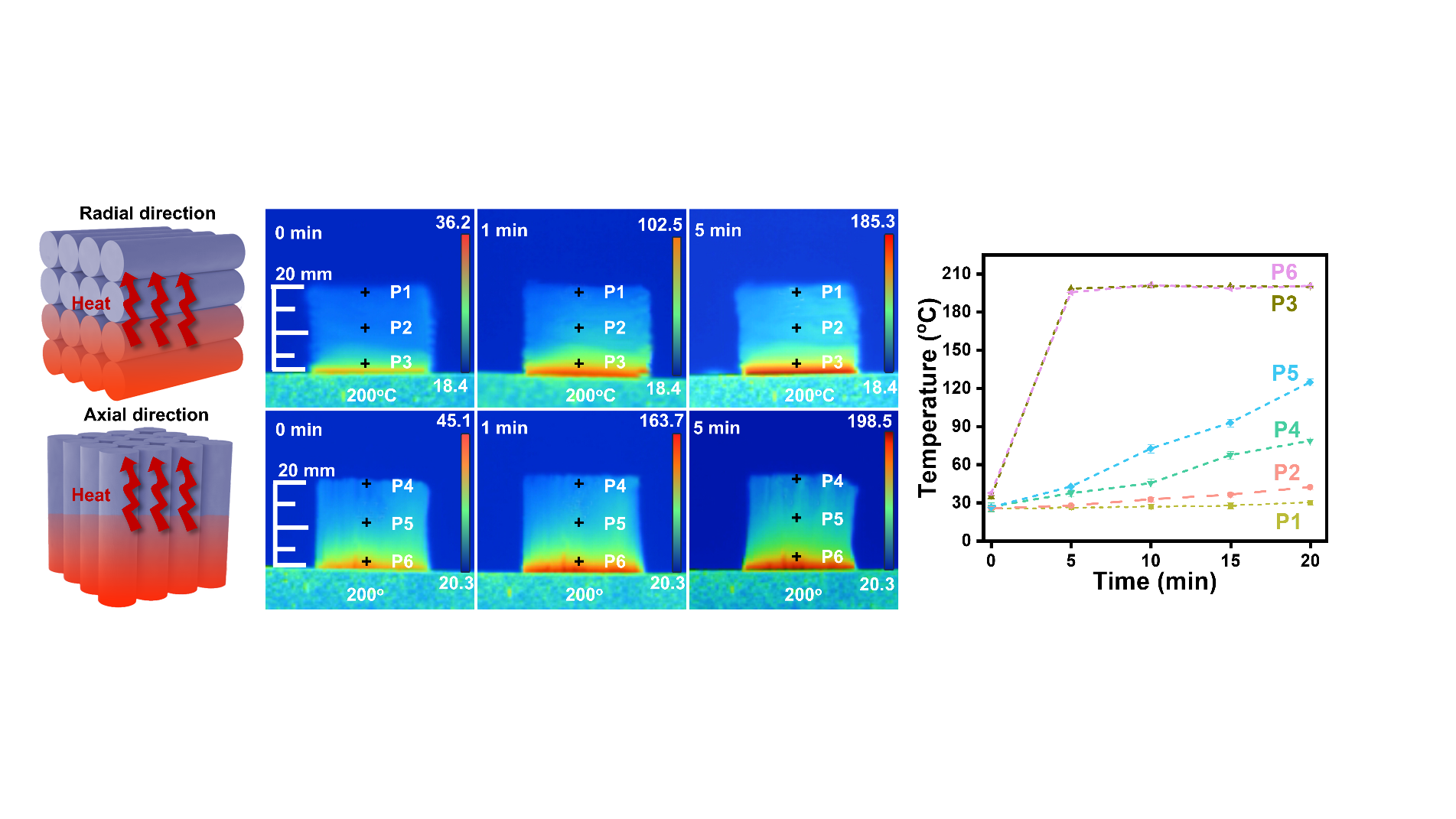


**Figure S7.** Model images and infrared thermograms of the A-SiC@SiO_2_-FAs placed radially and axially on a 200 ^o^C constant-temperature heating table, respectively, along with the temperature profiles at each observation point.

**Figure S8.** High-temperature thermal conductivity of anisotropic SiC@SiO_2_ fiber aerogels and isotropic SiC@SiO_2_ fiber aerogels.

**Figure S9.** FTIR spectra of untreated SiC nanofiber membrane and the A-SiC@SiO_2_-FAs.


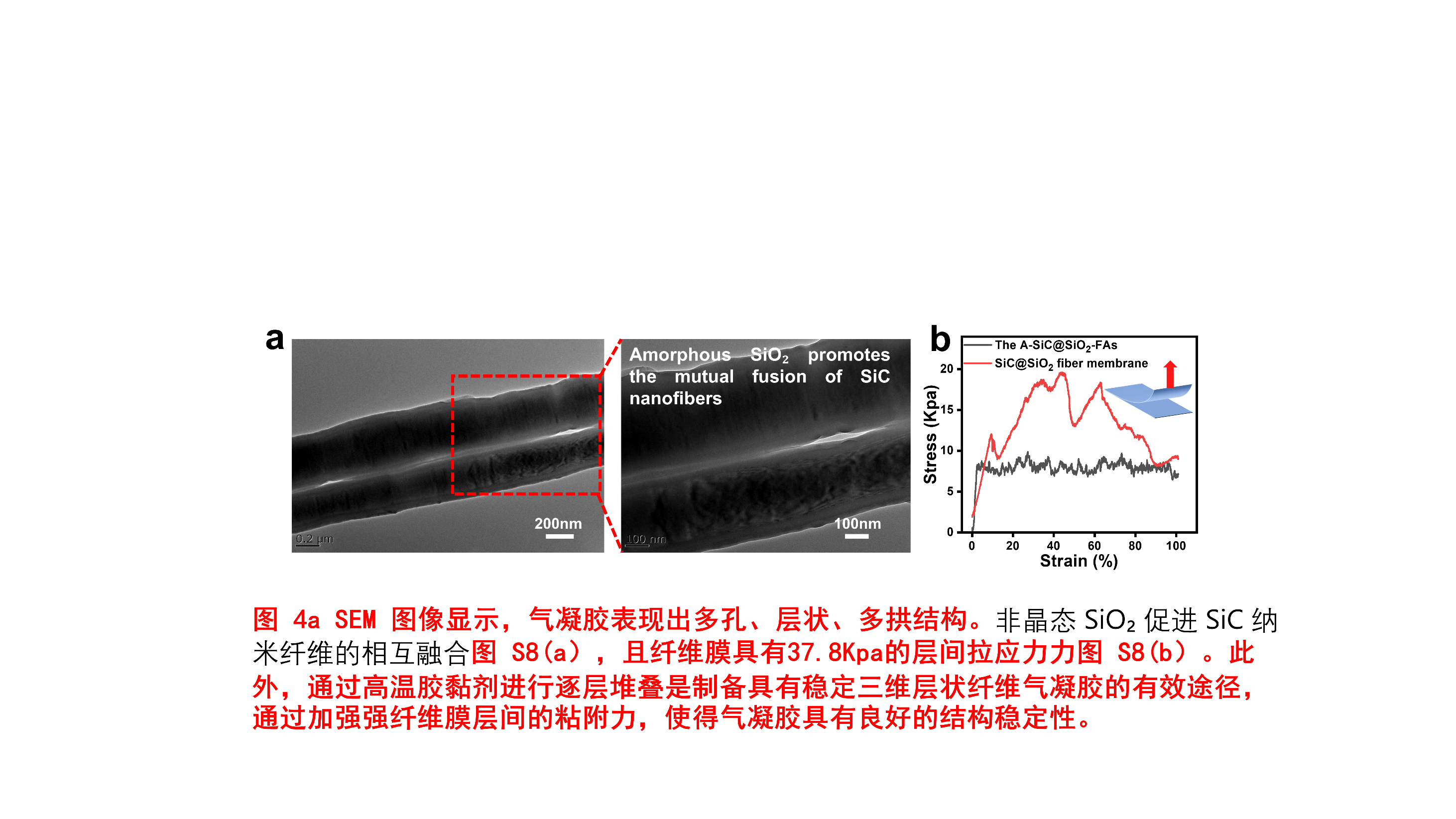


**Figure S110.** (a) TEM microstructure showing the SiC nanofibers are connected to each other by amorphous SiO₂. (b) The SiC@SiO_2_ fiber membrane interlayer adhesion test performance curve.


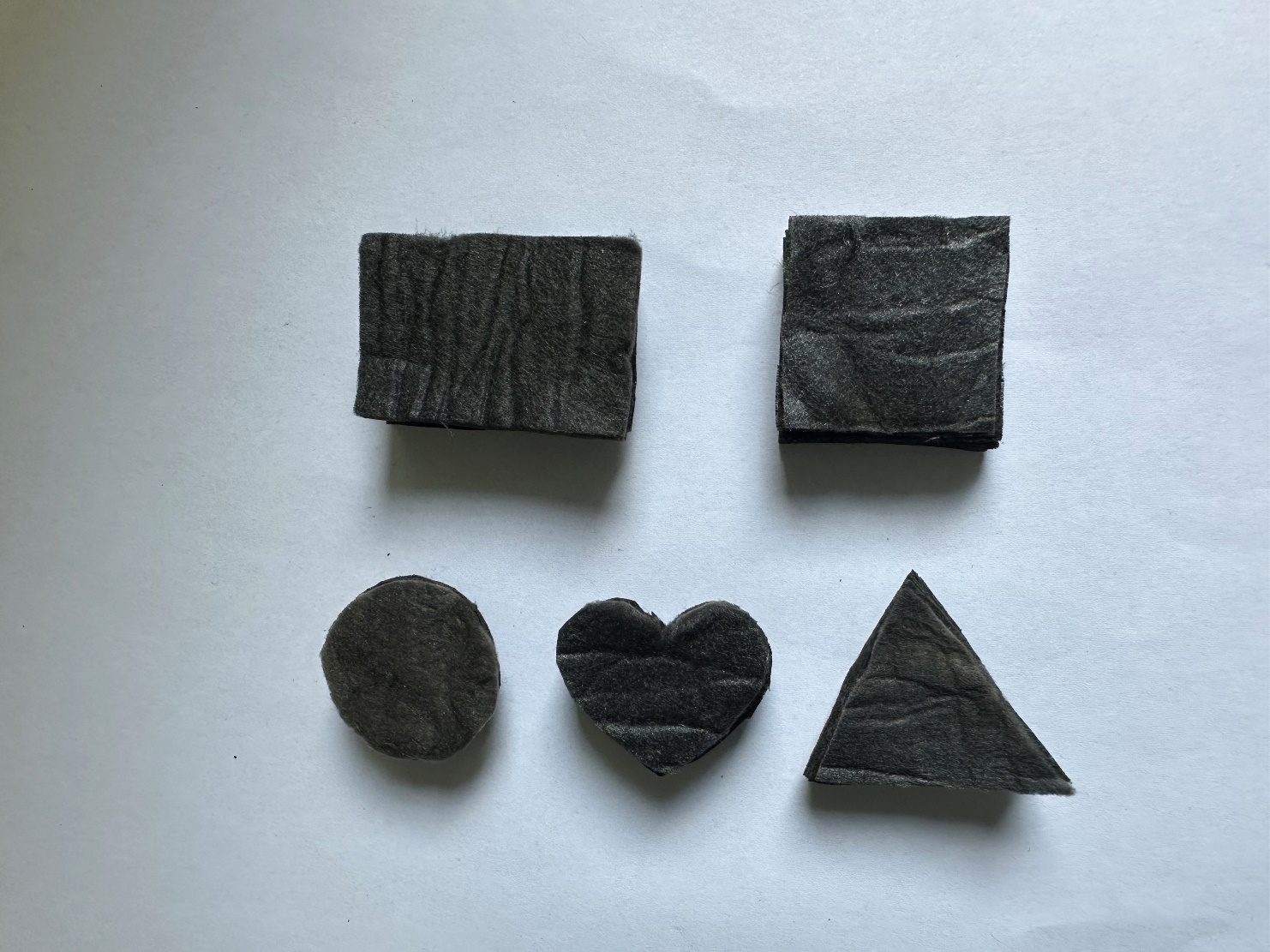


**Figure S11.** Optical images of the A-SiC@SiO_2_-FAs in various shapes.

**Figure S12.** Compression tests at different strain levels showed that the optimum recoverable deformation of the A-SiC@SiO_2_-FAs was 60%.


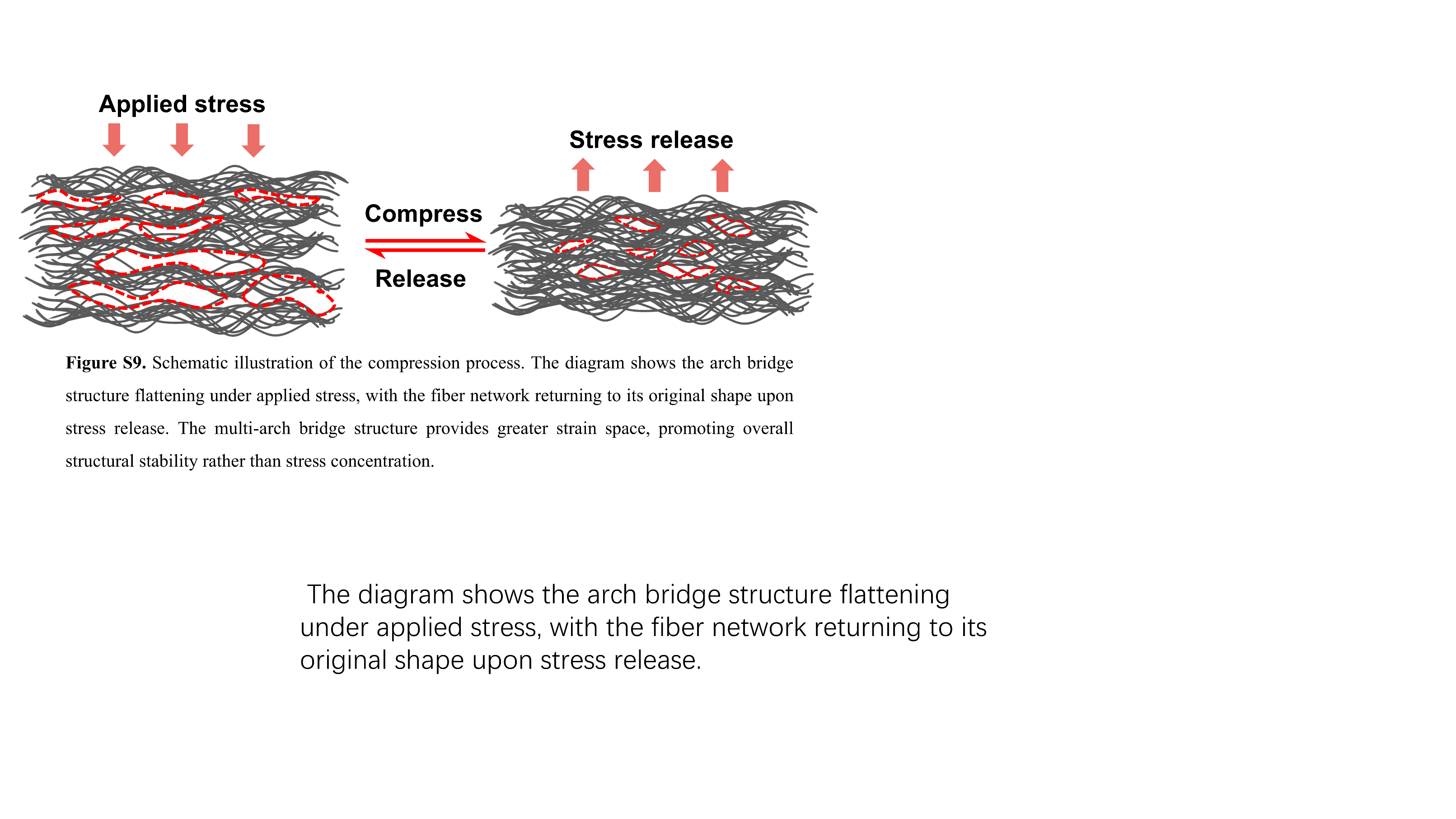


**Figure S13.** Schematic illustration of the compression process of the A-SiC@SiO₂-FAs. The diagram shows the arch bridge structure flattening under applied stress, with the fiber network returning to its original shape upon stress release. The multi-arch bridge structure provides greater strain space, promoting overall structural stability rather than stress concentration.

Thermal conductivity in the axial and radial directions was measured in air at room temperature by means of a Laser flash apparatus (LFA 457, NETZSCH, Germany). The thermal conductivity is measured by Equation (1):

$\kappa=\alpha\rho C_{p}$ (1)

$\kappa$ denotes the calculated thermal conductivity, $\alpha$ denotes the measured thermal diffusivity, $\rho$ denotes the density of the aerogel, and $C_{p}$ is the specific heat capacity. $C_{p}$ was measured by (DSC 2500, TA, USA) and the detailed results are shown in Table **S1**.

**Table S1.** Detail results of the calculated radial and axial thermal conductivity of the A-SiC@SiO_2_-FAs at room temperature.

| **Direction** | **Thermal diffusivity (mm^2^ s^-1^)** | **Density (mg cm^-3^)** | **Specific heat capacity (J g^-1^ K^-1^)** | **Thermal conductivity (mW m^-1^ K^-1^)** | **Std. Dev.** |
| --- | --- | --- | --- | --- | --- |
| Radial | 0.8437 | 27 | 0.791 | 18.02 | 0.013 |
|  | 0.8438 |  |  | 18.02 | 0.012 |
|  | 0.8461 |  |  | 18.07 | 0.012 |
| Axial | 4.2801 |  |  | 91.41 | 0.0312 |
|  | 4.2782 |  |  | 91.37 | 0.0312 |
|  | 4.2796 |  |  | 91.40 | 0.0312 |

**Table S2.** The list of thermal conductivity and anisotropy factors of anisotropic aerogel materials and corresponding references

| **Radial Thermal conductivity**  **(mW m^-1^ K^-1^)** | **Axial Thermal conductivity (mW m^-1^ K^-1^)** | **Anisotropy factor** | **References** |
| --- | --- | --- | --- |
| 14 | 35 | 2.5 | ^[1]^ |
| 26 | 35 | 1.35 | ^[2]^ |
| 30 | 60 | 2 | ^[3]^ |
| 29.6 | 37.8 | 1.28 | ^[4]^ |
| 28 | 120 | 4.29 | ^[5]^ |
| 27 | 34 | 1.26 | ^[6]^ |
| 23 | 44 | 1.91 | ^[7]^ |
| 27.75 | / | ~4 | ^[8]^ |
| 37 | 57 | 1.54 | ^[9]^ |
| 53.7 | / | 0.98 | ^[10]^ |
| 37.2 | 41.8 | 1.12 | ^[11]^ |
| 18 | 91.4 | 5.07 | This work |

**Movie S1.** Movie of compression release of the A-SiC@SiO_2_-FAs under liquid nitrogen environment.

**Movie S2.** Movie of A-SiC@SiO_2_-FAs heated under a butane torch for 1h: video at 60x speed.

**References**

[1] L. Su, H. Wang, M. Niu, S. Dai, Z. Cai, B. Yang, H. Huyan, X. Pan, *Sci. Adv.* **2020**, *6*, eaay6689.

[2] L. Li, Y. Zhou, Y. Gao, X. Feng, F. Zhang, W. Li, B. Zhu, Z. Tian, P. Fan, M. Zhong, H. Niu, S. Zhao, X. Wei, J. Zhu, H. Wu, *Nat. Commun.* **2023**, *14*, 5410.

[3] T. Li, J. Song, X. Zhao, Z. Yang, G. Pastel, S. Xu, C. Jia, J. Dai, C. Chen, A. Gong, F. Jiang, Y. Yao, T. Fan, B. Yang, L. Wågberg, R. Yang, L. Hu, *Sci. Adv.* **2018**, *4*, eaar3724.

[4] J. Luo, Y. Wang, Z. Qu, W. Wang, D. Yu, *Chem. Eng. J.* **2022**, *442*, 136388.

[5] J. Song, C. Chen, Z. Yang, Y. Kuang, T. Li, Y. Li, H. Huang, I. Kierzewski, B. Liu, S. He, T. Gao, S. U. Yuruker, A. Gong, B. Yang, L. Hu, *ACS Nano* **2018**, *12*, 140.

[6] J. Tong, H. Gao, Y. Weng, Y. Wang, *Macromol. Mater. Eng.* **2023**, *308*, 2200538.

[7] X. Zhang, X. Zhao, T. Xue, F. Yang, W. Fan, T. Liu, *Chem. Eng. J.* **2020**, *385*, 123963.

[8] Y. Yang, B. Dang, C. Wang, Y. Chen, K. Chen, X. Chen, Y. Li, Q. Sun, *Adv. Funct. Mater.* **2023**, *33*, 2307242.

[9] J. Garemark, J. E. Perea-Buceta, D. Rico Del Cerro, S. Hall, B. Berke, I. Kilpeläinen, L. A. Berglund, Y. Li, *ACS Appl. Mater. Interfaces* **2022**, *14*, 24697.

[10] X. Li, S. Li, Q. Wen, Y. Wan, L. Yang, Y. Kong, Y. Liu, S. Tian, C. Ma, *J. Eur. Ceram. Soc.* **2023**, *43*, 5606.

[11] C. Xie, L. He, Y. Shi, Z.-X. Guo, T. Qiu, X. Tuo, *ACS Nano* **2019**, *13*, 7811.
